# Design and Development of a Suite of Intimate Partner Violence Screening and Safety Planning Web Apps: User-Centered Approach

**DOI:** 10.2196/24114

**Published:** 2021-12-21

**Authors:** Patricia O'Campo, Alisa Velonis, Pearl Buhariwala, Janisha Kamalanathan, Maha Awaiz Hassan, Nicholas Metheny

**Affiliations:** 1 MAP Center for Urban Health Solutions St. Michael's Hospital Toronto, ON Canada; 2 Division of Community Health Sciences University of Illinois at Chicago School of Public Health Chicago, IL United States; 3 School of Nursing and Health Studies University of Miami Coral Gables, FL United States

**Keywords:** intimate partner violence, web-based applications, women, user-centered design

## Abstract

**Background:**

The popularity of mobile health (mHealth) technology has resulted in the development of numerous apps for almost every condition and disease management. mHealth and eHealth solutions for increasing awareness about, and safety around, intimate partner violence are no exception. These apps allow women to control access to these resources and provide unlimited, and with the right design features, safe access when these resources are needed. Few apps, however, have been designed in close collaboration with intended users to ensure relevance and effectiveness.

**Objective:**

The objective of this paper is to discuss the design of a suite of evidence-based mHealth and eHealth apps to facilitate early identification of unsafe relationship behaviors and tailored safety planning to reduce harm from violence including the methods by which we collaborated with and sought input from a population of intended users.

**Methods:**

A user-centered approach with aspects of human-centered design was followed to design a suite of 3 app-based safety planning interventions.

**Results:**

This review of the design suite of app-based interventions revealed challenges faced and lessons learned that may inform future efforts to design evidence-based mHealth and eHealth interventions.

**Conclusions:**

Following a user-centered approach can be helpful in designing mHealth and eHealth interventions for marginalized and vulnerable populations, and led to novel insights that improved the design of our interventions.

## Introduction

In recent years, web-based and mobile health (mHealth) technology has expanded greatly, providing a unique space for individuals to access information and resources to manage and improve health and well-being [1-3]. With upward of 5 billion individuals subscribing to wireless mobile services globally [4], digital space allows those seeking information or support to do so in a user-controlled, discreet, and accessible way [5-7]. This technology fills a unique gap for survivors of intimate partner violence (IPV), giving control to individuals themselves to access information about safety and resources. mHealth tools can be used at any time and any place, and survivors can return to the information as many times as needed to explore their concerns and their options as their situations change. In the past 20 years, more than 300 mobile or web-based apps for IPV have emerged [3,8], but only few have achieved widespread adoption. One limitation of apps that have failed to gain traction is their inability to meet the needs of specific communities or targeted groups, including the identification of local and appropriate resources.

The Partner Violence Implementation Science group is a collaboration between researchers, service providers, and survivors of IPV; our work is focused on cocreating interventions supporting individuals’ safety and self-determination. As part of these efforts, we identified the need for electronic IPV screening and safety planning resources tailored to the communities in which we work [3,4]. While a number of apps are available to assist women with identifying IPV and formulating safety plans, our community partners were concerned that many did not adequately address the nonlinear and multiphase process many women go through in an effort to increase safety for themselves and their children [9-11]. Gaining awareness of the possible dangers involved with taking action is one of these challenges, and women who have experienced IPV often lament that this awareness—along with information about how to increase relationship safety—did not come earlier in the process [12-14]. While our initial intent was to develop a tool to aid health care providers screen for IPV in an outpatient orthopedic clinic, our community partners—including survivors of IPV—felt that a web-based mHealth tool that was easy and quick to use, exclusively focused on identifying “red flags” for abusive behaviors, and that could help users identify safety options tailored for their situation was a critical need across settings. While we recognize that IPV is experienced by persons of all genders, our research focused on the experiences of the largest group of those experiencing IPV: cisgender women who are in relationships with cisgender men.

First, we looked at a variety of screening apps aimed at helping women identify if they are in an abusive relationship. After previewing a variety of publicly available screening apps across North America, we discovered that these tools often left out 1 or more types of violence, or did not use validated screening questions to detect IPV [15,16]. This led to our decision to create our own screening app that could help a wide range of individuals recognize patterns of unsafe behaviors in their own or others’ relationships and immediately access information about abuse and local services.

Next, for the smaller subset of women who may be experiencing behaviors related to IPV, a separate app was needed that could help them assess the severity of their situation (including the risk for lethality) and would promote safety planning behaviors tailored for their situations. One existing app provided much of what we were looking for. Developed by researchers at Johns Hopkins University [17-19] and tailored for Canadian audiences by researchers at Western University [20,21], the MyPlan app is an evidence-based mHealth app aimed at increasing users’ understanding of potentially dangerous patterns in their relationships reducing their ambivalence, or decisional conflict, about acting to alter these patterns and has been found to reduce future IPV in international randomized control trials [17,20,22-24]. After engaging with our target population and consultation with the teams at Johns Hopkins University and Western University, we decided to build on this evidence base and strong foundation to address additional needs present in our populations of interest as identified by our community partners in the Greater Toronto Area (GTA): women who were looking for more information about IPV, the majority of whom are not currently experiencing any unsafe relationship behaviors, and a second population of women who are living with IPV and need information about the potential for serious consequences of the abuse (up to and including lethality) and access to tailored safety planning that references geographically and culturally relevant strategies and resources.

This paper describes the rigorous process we engaged in to develop 3 evidence-based apps, each of which was developed for a particular audience and tailored to their specific needs. Using elements of human-centered design (HCD) [25], we ensure the end users (women at risk for IPV and those experiencing IPV) are involved in all stages of design and testing of our suite of mHealth tools. This includes iterative elicitation regarding the quality of experience, feelings of safety using the apps, and perceived helpfulness of the tools themselves throughout the design and testing processes [26,27]. Very few IPV screening or safety planning apps provide detailed information about the development or testing of the product, and there is scant peer-reviewed evidence regarding the process of developing the app, user testing, and evaluation [8]. Because of the critical role these tools can play for women who are concerned about the safety of themselves or a loved one, ensuring that the end product both addresses the primary concerns of women in a local community and uses evidence-informed processes to provide knowledge or bring about change is essential. Thus, we begin to shed light on this process by describing our iterative, community-engaged approach to building 3 separate, yet complementary, IPV mHealth interventions: an IPV screening tool (WithWomen), an individualized safety planning web app (Pathways), and the rapid adaptation of this app to the realities of living with IPV during COVID-19 (Promoting Safety in Emergencies, or PROMiSE). This suite of apps is summarized in Table 1.

## Methods

### Approach

Our approach to the design and adaptation of our suite of apps relied on the formation of partnerships with health care providers working to implement a screening program into their outpatient clinic setting, as well as service providers at IPV shelters, counseling groups for women with lived experience of IPV, and a peer support network for women living with IPV. We felt it important to keep these perspectives present throughout our design and adaptation process. Our partners played many roles: they advised us on key design features and content, connected us with women with lived experience who would provide feedback on the screening questions and functionality of the web apps, informed us of resources within the region which should be listed in the web apps, and connected us with plain language experts to ensure the content was appropriate for our population. The process used to develop each of these is discussed below. All 3 apps were approved by the St. Michael’s Hospital Research Ethics Board (REB # 15-361). Table 1 outlines the components of our suite of apps, their target populations, and main innovations, while Table 2 reviews the demographic characteristics of samples used to develop and test each of the mHealth tools.

The demographic characteristics of those who participated in the various surveys and interviews to inform the development of our screening and safety decision-support app are presented in Table 2.

**Table 1:** Partner Violence Implementation Science app suite components.

App feature	WithWomen Screener	Pathways	PROMiSE^a^
Target population	Women in relationships with men	Women in male–female relationships who have moderate-to-high safety concerns in their relationships	Women in male–female relationships who have moderate-to-high safety concerns in their relationships during public health emergencies
Purpose	Screen for potential IPV^b^	Safety planning and local resource connection	Modified safety planning and local resource connection during public health emergencies
Year of release	2018	2019	2020

^a^PROMiSE: Promoting Safety in Emergencies.

^b^IPV: intimate partner violence.

**Table 2:** Demographic characteristics of research participants^a^.

Demographics		Screening app				Pathways app			PROMiSE^b^ app	
		Cognitive interviews (n=18), n (%)	Anonymous encounter surveys (n=16), n (%)	App user testing (n=41), n (%)	Staff: preliminary user testing (n=19), n (%)		Clients: user testing (n=46), n (%)	User testing (n=7), n (%)		
**Age, years**										
	16-34	5 (28)	9 (56)	18 (44)	8 (42)		11 (24)	3 (43)		
	35-55+	13 (72)	7 (44)	22 (54)	11 (58)		35 (76)	4 (57)		
**Born in Canada and Indigenous**										
	Yes: Indigenous	0 (0)	1 (6)	4 (10)	4 (20)		8 (17)	3 (43)		
	Yes	10 (56)	9 (50)	17 (41)	8 (42)		16 (35)	0 (0)		
	No	8 (44)	7 (44)	20 (49)	10 (53)		21 (46)	4 (57)		
**Experiences of IPV^c^ in the past 5 years by the participant or someone close to them**										
	**Yes**	9 (50)	Not asked	31 (76)	13 (68)		39 (85)	2 (28)		
	**No**	8 (44)	Not asked	10 (24)	6 (32)		7 (15)	5 (72)		

^a^Numbers do not always total to 100% due to missing responses for selected categories.

^b^PROMiSE: Promoting Safety in Emergencies.

^c^IPV: intimate partner violence.

### Developing the WithWomen Screening App

#### Selecting the Screening Questions

We sought to have valid and reliable screening questions. After reviewing multiple validated IPV screening tools and the peer-reviewed literature [28], we selected the *Hurt, Insult, Threaten, and Scream* (HITS) instrument to serve as the base for our screening app. The HITS instrument is brief, has been used in general practice and emergency department settings [29,30], demonstrated acceptable sensitivity (88%) and specificity (range 86%-97%), and has been extensively tested within different populations (eg, tested in women/men, Hispanic and African American women as well as in Spanish) [31,32]. However, one of the limitations of HITS is its inability to identify subtle experiences of IPV, such as coercive and controlling behaviors, as well as experiences of sexual IPV. We identified several additional questions by consulting the peer-review literature on validated screening questions concerning coercive control and through conversations with our community partners (eg, “partner controlling what you wear”).

To be sure we were identifying those app users with the greatest need for immediate assistance, we limited the recall period to the past 12 months and asked questions in terms of frequency, rather than only identifying if behaviors had happened at all. Additionally, we intentionally asked about both short-term (eg, dating) and long-term (eg, partners) relationships.

#### Testing the Questions

To ensure our screening questions were acceptable and appropriate, we sought input from a cross-section of women. We conducted anonymous encounter surveys with women in local shopping malls. We approached women in the food courts, explained our research, and asked if they had 10 minutes to complete a short survey. We asked women to give us feedback on the clarity and acceptability of the questions and whether we were missing questions about a particular type of violence. We also consulted with women who were more likely to have lived experience of IPV by posing the same questions to female clients who accessed services at our partner community agencies (eg, women’s shelters, organizations serving women involved with the justice system). We also consulted service providers who serve women more generally such as at health clinics or agencies who help women with employment or housing to seek their input.

Next, as we narrowed the set of candidate questions, we conducted 3 cycles of cognitive interviews to ensure the clarity of the questions [33]. These were face-to-face surveys asking women to tell us in their own words what each question was asking and to get their advice on any terms or phrases that were hard to understand or inappropriate. We revised the questions based upon feedback we received in between each cycle. We also sought to test the reliability of our questions by administering the questions to a subset of women and contacting them 1 month later to retake the survey. Agreement between test and retest was assessed through computing the intraclass correlation coefficient (ICC). We also established evidence for convergent validity with questions capturing safety-related activities such as talking to a counselor about relationship concerns or missing work due to relationship issues [34].

The final screening instrument consisted of 9 items. The next step was to assign a level of risk to an individual’s summed score on the full screening instrument. To do this, we first assigned a value of risk for the answers to each question. Using information on the severity of the violence described in the question and corroborating it with existing severity ratings for similar questions [17,32], we assigned risk values to each question, with the lowest value of risk being 0 and the highest level of risk being 3. For example, if the respondent’s partner, ex-partner, or someone the respondent dated insulted her frequently, she received a question-specific level of risk of 2. On the physical violence item, she automatically received a question-specific score of 3 if she experienced any amount of beating, punching, kicking, strangling, or harm with a weapon. A sum score of 0-4 across all 9 questions indicates few to no safety concerns, 5-8 indicates there may be safety concerns, and 9-24 indicates that there are moderate to high safety concerns in the relationship (see Textbox 1 for scoring summary).

#### Finalizing Our Screening Questions

Using feedback from the cognitive interviews, we made slight wording changes to our questions to ensure their acceptability to women. For example, the question on sexual violence originally featured the word “coerce”; however, not all women interviewed understood the meaning behind the word and it was changed to “pressure, threaten, or force.” Overall, the questions were highly acceptable and clear to potential users, largely due to the fact that we relied on previously validated screening tools as the source of our questions. Almost perfect test–retest agreement was demonstrated via the 16 reliability interviews (ICC 96%; 95% CI 90%-99%). Reliability was similar when the sample was reduced to only those women who reported at least one positive answer to the screening questions (ICC 95%; CI 79%-99%).

**WithWomen final rapid intimate partner violence screening questions with points assigned to answers to reflect level of safety risk in a relationship.**

1. Over the last 12 months, how often did you feel uncomfortable doing or saying things around your current partner or someone you’re currently dating?

0=never, rarely, sometimes

1=frequently

2. Over the last 12 months, how often did your partner, an ex-partner, or someone you dated INSULT you or talk down to you?

0=Never, rarely

1=Sometimes

2=Frequently

3. Over the last 12 months, how often did your partner, an ex-partner, or someone you dated yell, shout, or curse at you?

0=Never, rarely

2=Sometimes

3=Frequently

4. Over the last 12 months, how often did your partner, an ex-partner, or someone you dated control who you see, where you go, what you do, or what you wear?

0=Never

2=Rarely, Sometimes

3=Frequently

5. Over the last 12 months, how often did your partner, an ex-partner, or someone you dated make you feel afraid or scared of them?

0=Never

2=Rarely, sometimes

3=Frequently

6. Over the last 12 months, how often did your partner, an ex-partner, or someone you dated THREATEN to harm you or someone you care about?

0=Never

2=Rarely

3=Sometimes, Frequently

7. Over the last 12 months, how often did your partner, an ex-partner, or someone you dated physically HURT you?

0=Never

2=Rarely

3=Frequently, Sometimes

8. Over the last 12 months, how often did your partner, an ex-partner, or someone you dated beat, punch, kick, strangle, or hurt you with a weapon?

0=Never

3=Rarely, Sometimes, Frequently

9. Over the last 12 months, how often did your partner, an ex-partner, or someone you dated force, threaten, or pressure you to participate in any sexual activity when you didn’t want to?

0=Never

3=Rarely, Sometimes, Frequently

Points for each question are summed and categorized into the following categories. The results screen displays the color (eg, yellow) and the explanation of the result (ie, “there are some things about your relationship that are of concern”) at the end of rapid screening.

0-4: Healthy (Green): There are few to no concerns regarding safety in your relationship

5-8: Caution (Yellow): There are some things about your relationship that are of concern.

9-24: Confirmed abuse (Red): Your relationship has many safety concerns.

**Figure 1:**
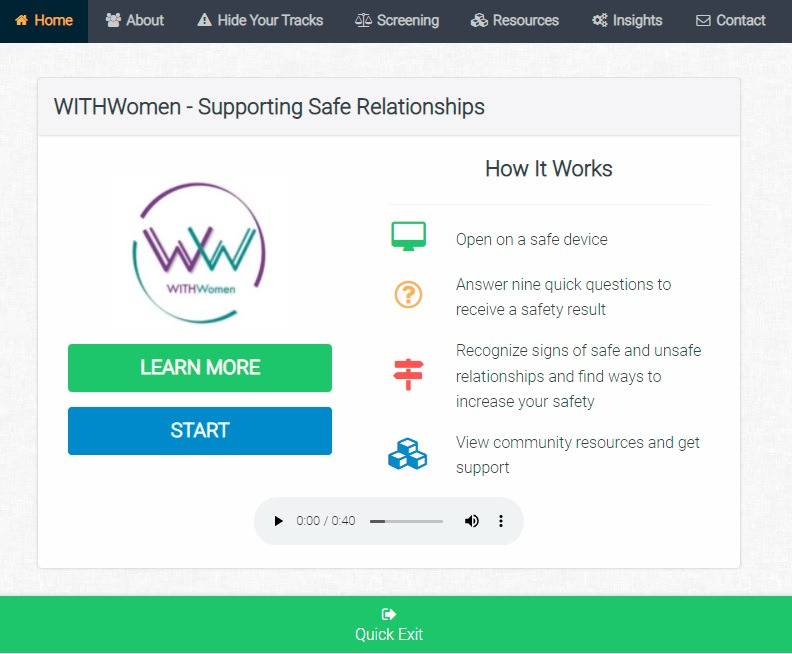
WithWomen Landing Page.

We established concurrent validity of our scales by correlating scores on the 9-item violence scale with responses to questions about whether women took part in activities that might be expected for women who were concerned about safety in their relationships: searched the internet for information about IPV; talked to a social worker or other professional or family and friends about her relationship; missed work because of relationship issues; or called the police due to relationship issues. Spearman correlations between the scores on the IPV screener and the 4 behaviors ranged from 0.72 to 0.82. We did ask about 1 more behavior, sought medical attention due to violence, and its correlation with our IPV screener was lower (0.57). Thus, concurrent validity was moderately high for all but 1 of the items, seeking medical care, but given that this activity happens relatively rarely and is only expected for 1 type of violence, physical violence, this level of correlation might be expected. The WithWomen landing page is shown in Figure 1.

**Figure 2:**
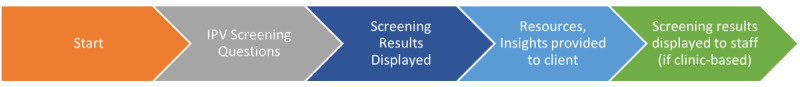
WithWomen User Journey.

The user journey for our WithWomen Screening app is presented in Figure 2, illustrating the simplicity and completeness of the app as it screens for IPV, provides an interpretation of the screening result, and provides links to resources related to prevention or management of IPV. Moreover, the app can be used to serve the public or be used in a clinic setting providing opportunities to share the screening result with clinic staff.

### Developing the Pathways Safety Planning App

#### Overview

As mentioned earlier, existing safety planning and decision-support apps did not meet the unique needs of our target population. In particular, we learned from our work with our community that the current apps required changes in terms of language, levels of interactivity, more flexibility in the user journey, accessibility of populations with literacy challenges, content, and safety features, and thus a new app was required. Since Pathways is intended to be a tool specifically for users who have self-identified as currently or recently experiencing IPV (ideally because they have used the WithWomen app), we designed Pathways to introduce users to the several options available in the app to begin making safety planning decisions. These options include (1) assessing severity, danger, and potential lethality of violent behaviors in their relationship; (2) identifying their safety priorities; (3) learning about safety actions to take that align with their priorities; and (4) learning about healthy and unhealthy relationships. We drew on existing safety planning and decision-support apps for IPV and made key modifications for our target population of urban Canadian women. These included tailoring the app to address women’s top concerns related to safety planning while still in or recently exiting a violent relationship. The goal of this app, then, is to allow women to prioritize their safety planning needs and receive tailored support, tangible action items, and referrals to local resources that match their highest concerns.

#### Step 1: Prototype Development

To develop the prototype for our app, we recruited 19 service providers and 31 women who identified as IPV survivors to interact with sections of the existing MyPlan app. In addition to this, we asked a smaller subset of participants with lived experience to review the full app online. Participants were recruited from 8 IPV-focused organizations in greater Toronto. We completed this user research over the course of 2 months, making note of their preferences, experiences with interacting with the existing app content and activities, and suggested modifications to inform the development of our Pathways app.

We then drew on 2 sources of data to determine the specific concerns most important to our users, which likely differed from the college-age females for whom the original MyPlan app was intended. We recruited 16 additional women who did not participate in the assessment of the existing safety planning app with different levels of lived experiences of IPV. Participants completed an anonymous survey that asked them to select how important 10 concerns are to women who are experiencing IPV as they make important decisions about their safety. In addition to those considered in the existing app, we reviewed the literature and consulted service provider key informants to arrive at these 10 concerns, which included having resources (finances, housing, legal services), privacy, immigration, and career-related concerns [13,35] (Table 3). When literacy was a challenge, a trained data collector supported respondents by reading out the instructions, explaining the activity, and writing down their responses. These answers were summed across participants and priorities that received the highest ratings were chosen as priority areas to which women could tailor their safety planning activities in Pathways.

**Table 3:** Relative ranking for each priority area for the My Concerns section of Pathways.

Rank	Safety priorities	Relative ranking
1	Having resources (finances, housing, legal support)	High
2	The health and well-being of someone close to you	High
3	Housing concerns	High
4	Privacy	High
5	Your personal health and well-being	Moderate
6	Language barriers	Moderate
7	Studies and career	Moderate
8	Immigration concerns	Moderate
9	Feelings for a partner	Low
10	Connections to the community	Low

#### Step 2: User Testing

**Figure 3:**
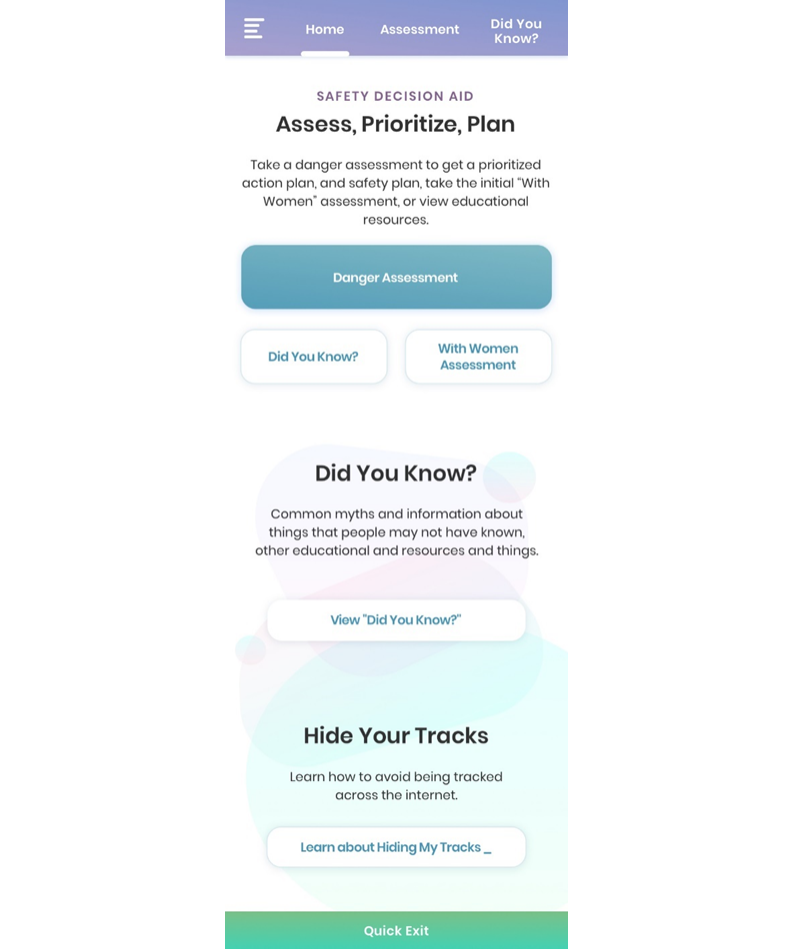
Pathways Landing Page.

Once the prototype data were collected, we worked with a software development firm, Tactica Interactive [36], to develop a working prototype of Pathways. Using this prototype and following HCD principles, we measured user experience with the app, looking specifically at functionality, ease of navigation, and the comprehensiveness of each of the tailored concern sections [26]. This process involved asking IPV survivors and service providers to engage with an online version of the app and provide feedback on usability of the different sections in an anonymous survey. A total of 26 individuals, including 5 IPV service providers, spent approximately 30-40 minutes reviewing all components of the app. User research was conducted at 4 IPV service organizations. One of the organizations provided trained interpreters to non-English speaking clients to make group activities more inclusive. As interpreters were already available in this setting, 5 women who spoke languages other than English were able to participate by working with an interpreter and a trained data collector to complete their survey responses. The user experience with Pathways is outlined in Figure 3.

#### Step 3: App Refinement

##### Finalizing Content for the Pathways App

For the section on user’s priorities, data from surveys conducted with participants and open-ended questions on what might be missing from app content shared with those taking part in the user research provided many options for what we might consider including in the prioritization section of the app.

Linking the safety options to a user’s priorities has been demonstrated to reduce decisional conflict about IPV safety planning [17,19,22]. In an effort to more closely align users’ concerns with their safety plans, “Resources” was further divided into “Finances,” “Housing,” and “Legal Support” as separate categories of Resource-based concerns. We removed “Child’s well-being” from the priority setting activity and instead incorporated a safety plan focused on children as a separate section in the Pathways app, where it could be given more prominence for those who select it.

The final 5 categories of concerns selected for inclusion in Pathways were each linked to an action plan, which suggests 3-6 specific actions users can take to address each area of concern. Safety steps associated with lower-ranked priorities were added into the optional viewing sections of the app. For example, suggestions to improve “Privacy” were included in the stalking-related safety plan, and also in the “Hide Your Tracks” section, which includes instructions for safe web browsing, while suggestions to address immigration concerns were added into the “Legal Support” section of the app. Algorithms were added to Pathways such that safety plans for the user’s most highly rated priority were presented to the user first.

##### Finalizing Pathways

Overall, user testing interviews confirmed the usefulness, understandability, appropriateness, and comprehensiveness of Pathways for our population. No major changes to safety planning–related content were suggested by respondents or our advisory groups. However, some common themes emerged about the language and structure of the app. For example, suggestions were made to simplify language, reduce text, and use check boxes whenever possible. In some sites, words such as “cue,” “priority,” and “prioritize” were not considered common language. We simplified the language and changed section titles (eg, “My Priorities” became “My Concerns” in Pathways). Several clients and service providers commented about the need to use reaffirming, rather than alarming language. Service providers also acknowledged the need to balance between providing validation and affirmations without normalizing severely dangerous behaviors. This was particularly relevant to the Danger Assessment section of the app. Our team removed questions that were in the Danger Assessment section but not used in the ratings of violence severity and prefaced the questions with plain language content explaining the rationale for asking them.

Several participants discussed the benefit of incorporating more tailored resources based on various needs of diverse populations. The recommendations were mostly around community supports specifically for minorities and people with language barriers, culturally sensitive services, and legal advice. Keeping in mind that language, transit access, and cost are barriers to accessing formal supports, we included links to support services that could be accessed by people free of charge or on sliding scale without the need for referrals or health insurance. In many cases, we listed support services in Pathways that are offered in multilingual, multicultural, or multisite service settings, and we included options to search resources by postal code whenever possible to allow women to find the most appropriate resources.

Another key area of concern pertained to the privacy, security, and accessibility of the app. To address these concerns, we incorporated a quick exit bar at the bottom of each app page that allows users to quickly hide their screen by redirecting them to the Google search engine. We also added a separate section in the app called “Online safety” that contains detailed information on secure web browsing. Our inclusive design intern redesigned the “Did You Know” section of our app, which originally described aspects of healthy and unhealthy relationships, into an activity where women interactively explore violence-related information to increase their knowledge about relationship violence. To increase accessibility for users with visual impairments or low literacy, we added an audio feature to each page of the app. The feature reads all text aloud and narrates navigation options on each screen.

The majority of the Pathways beta testers provided positive feedback (good or very good experience using the app) and noted that they would recommend the app to someone else. The lowest scored section of our survey was in relation to ease of navigation, with 15/46 (33%) of respondents identifying sections of the app that had too much text. To address these concerns, we took these sections to plain writing workshops hosted by our hospital’s patient education department, where plain language experts reviewed the material and provided feedback. We then hired an editor to incorporate suggestions and reduced the amount of text in flagged sections of the app by 20%. We also simplified the user flow, by moving certain sections of text to separate pages, and making those pages optional to view.

### Data for Our Apps

For safety reasons and also to honor concerns around privacy expressed by our informants with lived experience throughout the process of creating this suite of apps, we designed our apps to collect all data anonymously. Thus, no names, IP addresses, or any other identifiers are collected or stored. The research team does, however, collect other data for quality improvement purposes (eg, distribution of scores for screening or the danger assessment or priority concerns of those using Pathways or what time of day the apps are accessed), but we examine all data anonymously. We also ensured that no trace is left on any devices that have accessed any of our apps. The data are stored on servers in North America and the research team owns the data.

### Developing PROMiSE

The Pathways app was launched in December 2019. However, by early 2020 it was evident that the COVID-19 pandemic and the policies required to mitigate its spread (eg, stay-at-home orders, reduced service capacity) would lead to increased time spent in close quarters with abusive partners, widespread uncertainty, and financial strain, all stressors known to increase the risk for IPV [37]. Rates of IPV have indeed increased since the advent of the pandemic [38,39], and many women are unable to access IPV services due to reduced capacity and increased demand, making IPV screening and safety planning mHealth interventions critical to maximizing women’s safety during this and future public health emergencies. However, we received feedback from community partners that aspects of the Pathways app were not well suited to public health emergencies. For instance, the decision-support tools in Pathways are centered on seeking help outside the home and connecting with community services, many of which are either not open due to government restrictions, have closed due to financial strain, or are over capacity due to increased demand. It became clear that a safety planning tool was needed that responded to the realities women face during public health emergencies and that provides up-to-date, relevant information, and advises on actions to maximize safety in a safe, discreet way.

Using the same HCD-informed approach, we began a rapid research project to adapt Pathways for use during COVID-19 that involved (1) conducting a rapid systematic search of the peer-reviewed and gray literature on strategies that women experiencing IPV in the context of COVID-19 might find helpful and (2) convening an expert panel of IPV survivors and IPV service providers in the GTA to brainstorm new and modified strategies that women experiencing IPV in the context of COVID-19 might find helpful. This 3-month rapid research yielded 22 strategies that were either highly or somewhat recommended (eg, staying connected with others and planning for safety) and 6 that were not recommended (eg, hiding items that might be used as weapons) as they might make violence worse. Armed with new information about how women who are currently experiencing violence can maximize their safety during COVID-19, we connected again with Tactica to develop the new PROMiSE app from the existing Pathways app.

**Figure 4:**
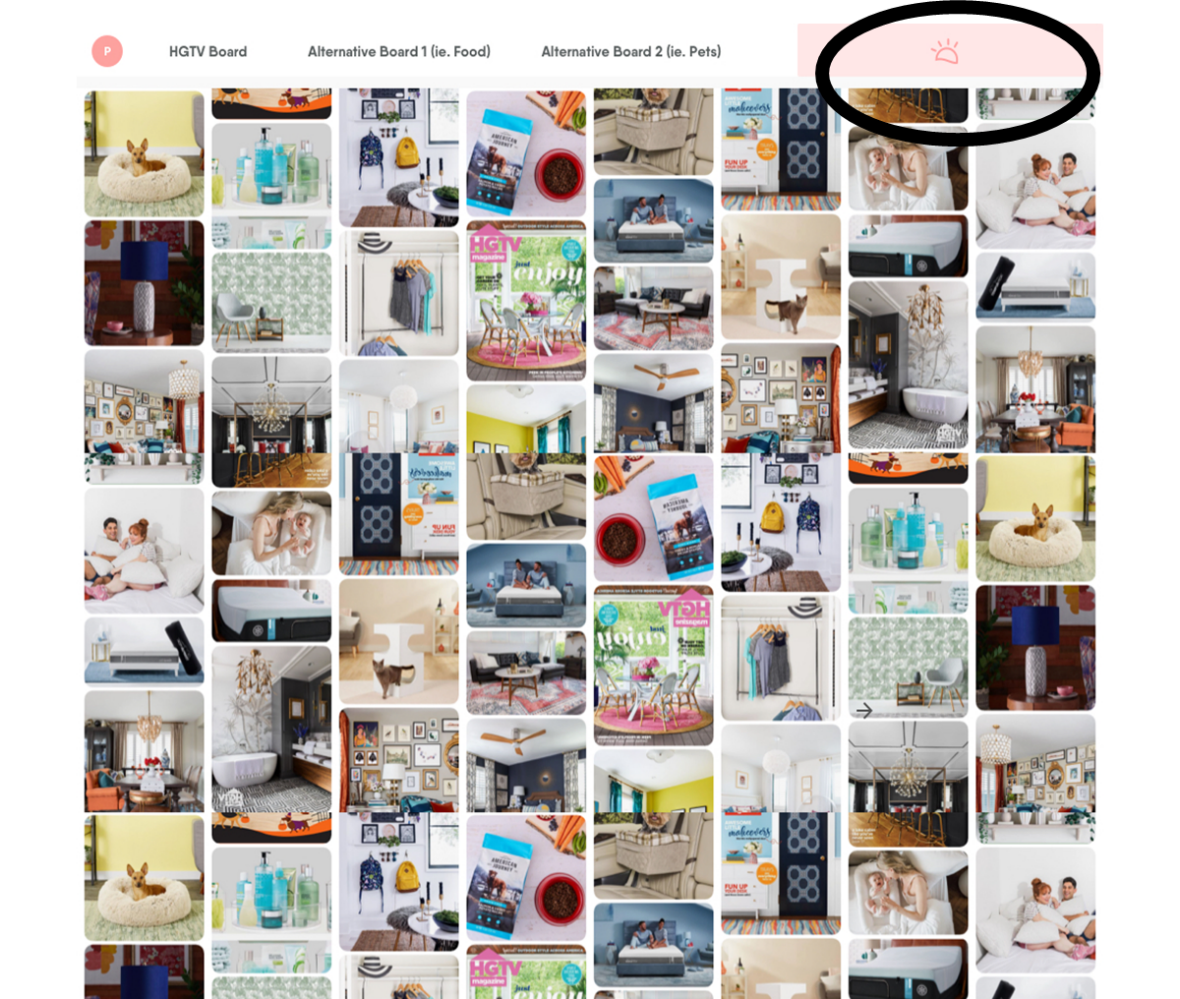
PROMiSE Landing Page as Disguised.

**Figure 5:**
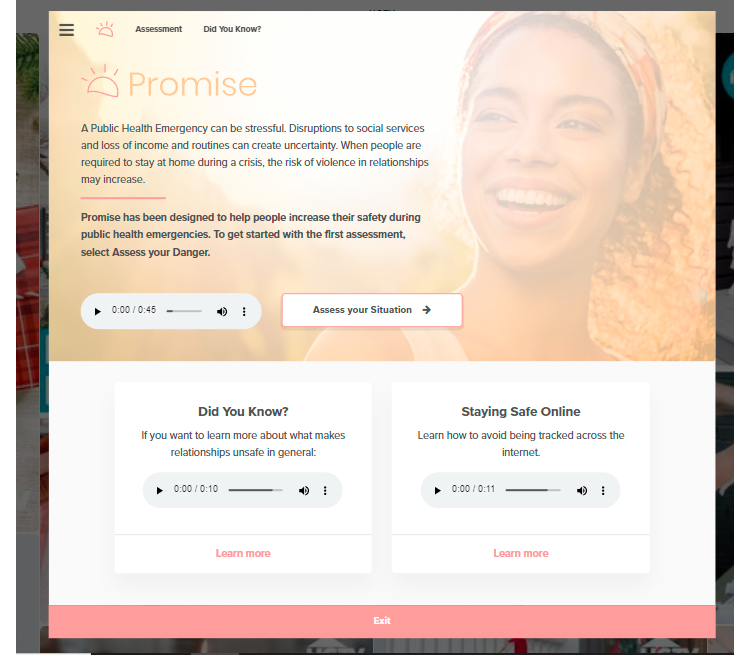
The actual PROMiSE landing page.

In addition to updating the content, we felt it was especially important to ensure the look and feel of the app was discreet, given that many more women may be using PROMiSE in close proximity to their abusers. To this end, we partnered with Tactica to develop a disguise feature for PROMiSE, wherein the app content is overlaid onto an innocuous webpage. We chose the main Pinterest board for Home and Garden Television as our innocuous landing page based on feedback from survivors of IPV that this would be unlikely to arouse suspicion from an abuser. When a woman visits the site, she will recognize the PROMiSE logo in the top right corner from advertisements and marketing materials (Figure 4). When this is clicked, the PROMiSE content will appear as a “pop-up” (Figure 5). In addition to the quick exit bar present in WithWomen and Pathways, users who click anywhere outside the “pop-up” box that contains the PROMiSE content will hide the app itself, revealing only the background website. The first time a woman visits the site, she will be offered an interactive tutorial that explains how to hide the content and make it reappear using hotkeys (desktop version) or a hotspot (on touchscreen devices).

Because the layout and technical aspects of PROMiSE are identical to those of Pathways, we forewent much of the technical user testing in service of a rapid rollout of this app in the midst of the pandemic. However, once a prototype of PROMiSE was available, we invited women with lived experience of IPV and IPV service providers to navigate the app and provide feedback on its new look and feel and to identify any glitches that may have occurred in the creation of the app (n=7; Table 2). This information will be incorporated into later updates of PROMiSE. PROMiSE was launched on December 1, 2020

## Discussion

### Lessons Learned

Web-based and mHealth apps are increasingly common for IPV [1,3]. This paper demonstrates how we built on the existing set of IPV screening and safety planning web-based apps to tailor and create a suite of tools appropriate for our local population of women who are at risk of or needing to plan for safety around IPV before and during COVID-19. Furthermore, given the range of preventive and educational activities taken up by web-based and mHealth IPV-related apps, we focused our efforts on 2 particular challenges: (1) helping women to learn early on about unsafe relationship behaviors; and (2) once violence is confirmed, tailored safety planning to maximize safety. These apps were designed to complement the vast set of existing resources and services in our metropolitan area but also serve to provide a unique resource by taking into account a woman’s particular priorities and being accessible any time of day and any place that the internet is available.

Systematic reviews of mHealth, computer-, and web-based apps have noted the scarcity of engagement with affected communities in the design of applications that impact the relevance and utilization of such apps [3]. We addressed this limitation in our work by using elements of HCD, which sought input from and cocreation with survivors of IPV and health care providers who work closely with this population. While this required that additional time be built into each of our phases of research, this increased the relevance of our apps to our target population. We found this to be critically important given the stage of IPV that we are targeting with these apps: for WithWomen screening we are targeting women who are yet unaware of the safety threats present in their relationships, and for Pathways and PROMiSE we are targeting women with varying levels of violence in their intimate relationships who are needing to take some kind of action to increase safety in their relationships. While an existing app [18] was the template for our Pathways and PROMiSE, our apps’ new features requested and endorsed by intended users included priorities that aligned with their preferences, interactive sections on healthy relationship information, freedom within the user journey to access any part of the app, plain language text, and an audio option.

We learned from survivors, providers, and also from the literature that abuse itself impacts the survivors’ trust in others and confidence in themselves for making sound decisions about their relationships and safety-related actions. Furthermore, despite the vast strength and resilience of survivors, their experiences with relationships where they are routinely admonished and denigrated by perpetrators result in pervasive survivor self-doubt and self-blame, further affecting information processing and decision making around safety [40-44]. Our user research and advice from our partners helped shape the language and tone of the text in the apps. Because of the engagement of women and health and social services providers in the process of undertaking user research, we employed affirmative and strength-based language and phrases. Moreover, given levels of self-doubt among survivors who are living with abuse, we also used language that simultaneously prioritized trusting users’ own instincts about maintaining safe behaviors while also encouraging safety planning action. One issue we were not able to overcome is the impact of missing data in our screening scales. If a respondent skips 1 or more questions, a value of 0 is used for the skipped question, potentially artificially deflating actual level risk.

Working closely with partners had other benefits. Our fracture clinic partner adopted a technology-enhanced approach to screening for IPV using our WithWomen IPV screening app. We used implementation science strategies to assist them with developing a suitable screening protocol that minimally impacted their busy clinic schedule [45] and for the first time ever the clinic had access to data about screening and IPV prevalence. While we did not plan for the broader use of our apps in service or health care settings, virtual visits with clients have significantly increased due to the pandemic and along with it the opportunity to incorporate technology-enhanced screening or safety planning during or in between client visits. With the release of PROMiSE, with its focus on safety planning during public health emergencies and enhanced safety features, we have been asked by providers to assist with the creation of options for including our apps within their virtual interactions with clients.

Because the key features of MyPlan that led to positive safety planning outcomes were retained in our Pathways app, we feel that another clinical trial demonstrating that use of the app over usual care is not warranted [19]. However, we are planning to use innovative single-case experimental study designs to demonstrate, on a much smaller sample of participants, that the PROMiSE app is safe to use when in the home and generates positive impacts on IPV knowledge and safety planning activities [46].

While we had evidence-based models to draw from, we discovered that creating these apps took far longer than we anticipated for several reasons. The iterative nature and multiple cycles of developing prototypes for sections of the apps, gaining feedback on those, and making modification to the prototypes take time. Our research team had to build capacity in some areas such as learning about the privacy concerns when our initial designs sought to link our rapid IPV screening app to the hospital data system and understanding the technical aspects of discreet app design. As we relied on the expert consultants and our hospital IT department in a set of iterative conversations about the app features that are or are not aligned with privacy requirements (eg, finding a secure way to link our app that is housed on external servers with the hospital-based electronic medical records), our process was further slowed as we were not a priority of the hospital’s IT concerns. Finally, we also experienced significant delays due to the initial technology development partner we had chosen. Our team initially engaged the services of a laboratory connecting clinicians with biomedical and computer engineering students to apply technological solutions to the real-world problems faced by clinicians in their practice. We were eager to give a student team the opportunity to build our app while simultaneously cutting down on development costs to accommodate our shoestring budget. Unfortunately, the combination of our lack of experience with developing an app, our inability to communicate the technological specifications to the student team, and our lack of understanding of how short or long the development of these apps should take resulted in a slow process that spanned almost 18 months that ultimately ended in failure. We probably erred by not including a member from the technology team in our discussions of the research process; however, rapid turnover in the student team would have posed another challenge to that strategy. Fortunately, when we subsequently turned to a professional app developer the work was completed in a matter of weeks. While we were fortunate enough to engage a Master’s student from the Inclusive Design program at the Ontario College of Arts and Design as an intern to advise on graphic design and user experience for WithWomen and Pathways, a diminishing overall budget precluded allowing us to have a designer or more advanced features to these aspects of our suite. Separate funding for PROMiSE, however, allowed us to make use of a professional designer and more technical consultants, resulting in increased functionality and advanced privacy features.

### Conclusion

With the use of technology in health and social service care intervention and delivery becoming more commonplace, there is an opportunity to develop high-quality IPV screening apps to implement in practice. The aim of this venture was to create a suite of IPV screening and safety planning apps for use with local women that contain relevant information and resources in a safe, discreet way. Evidence and user testing feedback have indicated what women want and need from IPV screening and safety planning apps: that is, the product to be relevant to its user population with easy navigation, HCD features, and the ability to access discreetly.

## Abbreviations

GTA: Greater Toronto Area
HCD: human-centered design
HITS: Hurt, Insult, Threaten, and Scream
ICC: intraclass correlation coefficient
IPV: intimate partner violence
mHealth: mobile health
PROMiSE: Promoting Safety in Emergencies

## References

[1] Goldstein KM, Zullig LL, Dedert EA, Alishahi Tabriz A, Brearly TW, Raitz G, Sata SS, Whited JD, Bosworth HB, Gordon AM, Nagi A, Williams JW, Gierisch JM (2018). Telehealth Interventions Designed for Women: an Evidence Map. J Gen Intern Med.

[2] Haddad SM, Souza RT, Cecatti JG (2019). Mobile technology in health (mHealth) and antenatal care-Searching for apps and available solutions: A systematic review. Int J Med Inform.

[3] Anderson EJ, McClelland J, Meyer Krause C, Krause KC, Garcia DO, Koss MP (2019). Web-based and mHealth interventions for intimate partner violence prevention: a systematic review protocol. BMJ Open.

[4] (2017). GSM Association.

[5] Aryana B, Brewster L (2020). Design for mobile mental health: Exploring the informed participation approach. Health Informatics J.

[6] Covolo L, Ceretti E, Moneda M, Castaldi S, Gelatti U (2017). Does evidence support the use of mobile phone apps as a driver for promoting healthy lifestyles from a public health perspective? A systematic review of Randomized Control Trials. Patient Educ Couns.

[7] Haggerty LA, Hawkins JW, Fontenot H, Lewis-O'Connor A (2011). Tools for screening for interpersonal violence: state of the science. Violence Vict.

[8] Eisenhut K, Sauerborn E, García-Moreno C, Wild V (2020). Mobile applications addressing violence against women: a systematic review. BMJ Glob Health.

[9] Burke JG, Mahoney P, Gielen A, McDonnell KA, O'Campo P (2009). Defining appropriate stages of change for intimate partner violence survivors. Violence Vict.

[10] Catallo C, Jack SM, Ciliska D, Macmillan HL (2012). Identifying the turning point: using the transtheoretical model of change to map intimate partner violence disclosure in emergency department settings. ISRN Nurs.

[11] Jane, Stoever (2013). Transforming Domestic Violence Representation. Kentucky Law Journal.

[12] Daoud N, Matheson FI, Pedersen C, Hamilton-Wright S, Minh A, Zhang J, O'Campo P (2016). Pathways and trajectories linking housing instability and poor health among low-income women experiencing intimate partner violence (IPV): Toward a conceptual framework. Women Health.

[13] Daoud N, Matheson FI, Pedersen C, Hamilton-Wright S, Minh A, Zhang J, O'Campo P (2016). Pathways and trajectories linking housing instability and poor health among low-income women experiencing intimate partner violence (IPV): Toward a conceptual framework. Women Health.

[14] DeKesseredy, Walter, Dragiewicz M, Schwartz M (2017). Abusive Endings: Separation and Divorce Violence Against Women.

[15] (2017). Purple Evolution Incorporated.

[16] (2019). Soujourner Peace App.

[17] Glass NE, Perrin NA, Hanson GC, Bloom TL, Messing JT, Clough AS, Campbell JC, Gielen AC, Case J, Eden KB (2017). The Longitudinal Impact of an Internet Safety Decision Aid for Abused Women. Am J Prev Med.

[18] Johns Hopkins University School of Nursing (2021). MyPlan App.

[19] Glass N, Eden KB, Bloom T, Perrin N (2010). Computerized aid improves safety decision process for survivors of intimate partner violence. J Interpers Violence.

[20] Ford-Gilboe M, Varcoe C, Scott-Storey K, Wuest J, Case J, Currie LM, Glass N, Hodgins M, MacMillan H, Perrin N, Wathen CN (2017). A tailored online safety and health intervention for women experiencing intimate partner violence: the iCAN Plan 4 Safety randomized controlled trial protocol. BMC Public Health.

[21] Ford-Gilboe M, Varcoe C, Scott-Storey K, Perrin N, Wuest J, Wathen CN, Case J, Glass N (2020). Longitudinal impacts of an online safety and health intervention for women experiencing intimate partner violence: randomized controlled trial. BMC Public Health.

[22] Eden KB, Perrin NA, Hanson GC, Messing JT, Bloom TL, Campbell JC, Gielen AC, Clough AS, Barnes-Hoyt JS, Glass NE (2015). Use of online safety decision aid by abused women: effect on decisional conflict in a randomized controlled trial. Am J Prev Med.

[23] Hegarty K, Tarzia L, Murray E, Valpied J, Humphreys C, Taft A, Gold L, Glass N (2015). Protocol for a randomised controlled trial of a web-based healthy relationship tool and safety decision aid for women experiencing domestic violence (I-DECIDE). BMC Public Health.

[24] Koziol-McLain Jane, Vandal AC, Wilson D, Nada-Raja S, Dobbs T, McLean C, Sisk R, Eden Karen B, Glass Nancy E (2018). Efficacy of a Web-Based Safety Decision Aid for Women Experiencing Intimate Partner Violence: Randomized Controlled Trial. J Med Internet Res.

[25] International Standards Organization (2010). Ergonomics of human-system interaction — Part 210: Human-centred design for interactive systems. ISO Standards Catalogue.

[26] Martínez-Pérez B, de LTI, Candelas-Plasencia S, López-Coronado M (2013). Development and evaluation of tools for measuring the quality of experience (QoE) in mHealth applications. J Med Syst.

[27] Harte R, Quinlan LR, Glynn L, Rodríguez-Molinero Alejandro, Baker PM, Scharf T, ÓLaighin Gearóid (2017). Human-Centered Design Study: Enhancing the Usability of a Mobile Phone App in an Integrated Falls Risk Detection System for Use by Older Adult Users. JMIR Mhealth Uhealth.

[28] Rabin RF, Jennings JM, Campbell JC, Bair-Merritt MH (2009). Intimate partner violence screening tools: a systematic review. Am J Prev Med.

[29] Spangaro J, Vajda J, Klineberg E, Lin S, Griffiths C, Saberi E, Field E, Miller A, McNamara L (2020). Intimate partner violence screening and response in New South Wales emergency departments: A multi-site feasibility study. Emerg Med Australas.

[30] Chen P, Rovi S, Washington J, Jacobs A, Vega M, Pan K, Johnson MS (2007). Randomized comparison of 3 methods to screen for domestic violence in family practice. Ann Fam Med.

[31] Chan C, Chan Y, Au A, Cheung G (2017). Reliability and Validity of the “Extended - Hurt, Insult, Threaten, Scream” (E-Hits) Screening Tool in Detecting Intimate Partner Violence in Hospital Emergency Departments in Hong Kong. Hong Kong Journal of Emergency Medicine.

[32] Sherin KM, Sinacore JM, Li XQ, Zitter RE, Shakil A (1998). HITS: a short domestic violence screening tool for use in a family practice setting. Fam Med.

[33] Collins D (2003). Pretesting survey instruments: an overview of cognitive methods. Qual Life Res.

[34] Wolford-Clevenger C, Kuhlman S, Elledge LC, Smith PN, Stuart GL (2019). A Preliminary Validation of the Suicidal Behavior Exposure Scale. Psychol Violence.

[35] Buel S (1999). Fifty obstacles to leaving, aka Why Women Stay. The Colorado Lawyer.

[36] Tactica International.

[37] Jewkes R (2002). Intimate partner violence: causes and prevention. Lancet.

[38] Godin M (2020). 2020 As cities around the world go on lockdown, victims of domestic violence look for a way out. Time Magazine.

[39] Dubinski K, Margison A (2020). National survey finds domestic violence during pandemic was more frequent and severe. Canadian Broadcasting Corporation.

[40] Matheson FI, Daoud N, Hamilton-Wright S, Borenstein H, Pedersen C, O'Campo P (2015). Where Did She Go? The Transformation of Self-Esteem, Self-Identity, and Mental Well-Being among Women Who Have Experienced Intimate Partner Violence. Womens Health Issues.

[41] Brosi M, Rolling E, Gaffney C, Kitch B (2019). Beyond Resilience: Glimpses into Women’s Posttraumatic Growth after Experiencing Intimate Partner Violence. The American Journal of Family Therapy.

[42] Anderson KM, Renner LM, Danis FS (2012). Recovery: resilience and growth in the aftermath of domestic violence. Violence Against Women.

[43] Lim BHP, Valdez CE, Lilly MM (2015). Making Meaning Out of Interpersonal Victimization: The Narratives of IPV Survivors. Violence Against Women.

[44] Ulloa Ec, Hammett JF, Guzman Ml, Hokoda A (2015). Psychological growth in relation to intimate partner violence: A review. Aggression and Violent Behavior.

[45] Velonis AJ, O'Campo P, Rodrigues JJ, Buhariwala P (2019). Using implementation science to build intimate partner violence screening and referral capacity in a fracture clinic. J Eval Clin Pract.

[46] O'Campo P, Mutaner C, Metheny N, Yakubovich A (2019). Improving experimental designs for social science research: refinements to the single case experimental design. Grant funded by the Social Sciences and Humanities Research Council (SSHRC). Social Sciences and Humanities Research Council of Canada.

